# The Impact of COVID-19 on the Mental Health and Wellbeing of Children with Special Education Needs and Disabilities: A Systematic Review

**DOI:** 10.1007/s40489-024-00453-2

**Published:** 2024-04-01

**Authors:** Victoria E. Castle, Vassilis Sideropoulos, Cat Jones, Dixiao Zhang, Jo Van Herwegen, Olympia Palikara

**Affiliations:** 1 Department of Education Studies, Faculty of Social Sciences, University of Warwick, Coventry, CV4 8UW UK; 2 Department of Experimental Psychology, University of Oxford, Oxford, England; 3 Department of Psychology and Human Development, IOE, UCL’s Faculty of Education and Society, London, England

**Keywords:** COVID-19, Special educational needs and disabilities (SEND), Mental health, Well-being, Systematic review

## Abstract

The COVID-19 pandemic has impacted greatly the mental health of children. We performed a systematic review to better understand the impact of the pandemic on children with special educational needs and disabilities (SEND) across different SEND categories. Following PRISMA guidelines, of 1699 search results, 66 studies were included in our analysis as they met our inclusion criteria concerning: a) children with SEND; b) focus on COVID-19; c) longitudinal or cross-sectional design; d) quantitative or qualitative measures; and e) mental health or well-being outcomes. Our review suggests that there was a typically negative impact on mental health and well-being for children with SEND, yet experiences varied on the basis of individual differences, rather than category of SEND. Findings highlight the need for interventions and policy implementations to improve the everyday mental well-being of this population.

The COVID-19 pandemic has had deleterious effects on the mental health of the general population across the globe including increased anxiety (Huang & Zhao, 2020), post-traumatic stress symptoms and an acute stress reaction (Liu et al., 2020), as well as increased suicidal ideation and attempts (Ammerman et al., 2021). The World Health Organization (WHO) define well-being as “a positive state experienced by individuals and societies” (World Health Organization, 2021, p. 16) directly impacting a person’s quality of life and the ability to contribute to and thrive in society. Mental health conditions can have a substantial and long-term impact on ‘normal day-to-day activities’ (UK Government, 2017). Mental well-being is considered to not just reflect the absence of a mental health condition, but also to encompass positive psychological experience (Agteren & Iasiello, 2020). Initial evidence has found a significant impact of the COVID-19 pandemic on child mental health (Chawla et al., 2021). Given the high comorbidity of mental health difficulties for those diagnosed with special educational needs and disabilities (SEND) prior to the COVID-19 pandemic (Rzepecka et al., 2011; Van Steensel et al., 2011), we might expect higher rates of mental health issues and lower mental well-being in SEND populations during the COVID-19 pandemic compared to the general population and compared to pre-pandemic levels.

Special Educational Needs and Disabilities (SEND) is an umbrella term that includes all individuals who have disabilities or individual differences that make learning via traditional classroom settings more difficult. This broad term includes those with neurodevelopmental conditions, such as Autism, Attention-Deficit Hyperactivity Disorder (ADHD), Intellectual Disability (ID), and specific learning difficulties, such as dyslexia and dyscalculia. The term also includes individuals with chromosomal disorders, such as Down syndrome and Williams syndrome. Finally, SEND can also be used to refer to individuals who experience difficulties with processing information due to birth trauma or early adversity. Although this broad term covers a range of difficulties, the connecting feature is that individuals with SEND all experience significant disadvantage for educational outcomes if teaching and assessment methods are not adapted to suit their individual needs (Timpson, 2014).

The COVID-19 pandemic has brought unpredictable and fast-paced changes to society, which have disproportionately impacted communities of children and young people with SEND as they are known to be more vulnerable and resistant to environmental change and disruptions to routines (Baweja et al., 2021; Kawaoka et al., 2022; Singh et al., 2020). The longevity of the COVID-19 pandemic has been unprecedented, and the uncertainty surrounding the end of both restrictions and risk to health has been challenging to many. What remains unclear, and in need of further exploration, is whether this effect is universal across SEND categories, and whether the pandemic has had a greater influence on one area of well-being over another (e.g. anxiety vs. mood).

The current review aims to assess the impact of COVID-19 on the mental health and well-being of children with SEND, by examining the change in mental health symptoms over the course of the COVID-19 pandemic, as reported in the literature. Researchers who attempted to provide clear pathways for the direction of research during COVID-19 have failed to focus on mental health in relation to children with SEND despite the emerging evidence highlighting the increased risk of mental health problems during the COVID-19 pandemic and the disruptions the pandemic has caused to families of children with SEND (Amaral & de Vries, 2020; Ameis et al., 2020; Baweja et al., 2021; Kreysa et al., 2022). However, Musa et al. (2021) highlight the need for a child/parent-centered approach that considers the mental health and well-being of children with SEND during the pandemic as there is a variation of how it impacted families of children with SEND, specifically autistic children. Therefore, the current review will be the first one to explore the impact of COVID-19 on mental health and well-being of not only autistic children, but of children with SEND across the board.

Unlike previous reviews and commentaries that have explored the impact of COVID-19 on mental health and well-being for autistic individuals (Ameis et al., 2020; Baweja et al., 2021; Kreysa et al., 2022; Musa et al., 2021), the current review aims to systematically review literature across SEND categories, in an attempt to elucidate whether impact on mental health/well-being has been limited to specific groups of children with SEND or whether this effect is universal across SEND categories. This systematic review will further explore data from both longitudinal and cross-sectional studies, using quantitative and/or qualitative designs, that explore the nature of the change in mental health or well-being for this group. By including a wide range of studies, this review can provide a clear overview of the literature base as a whole. It is further possible to examine whether certain methodological or sample characteristics influence the reported findings of studies, as well as whether these factors played distinct roles in the prediction of different observed mental health or well-being outcomes.

It is expected that children with SEND had poorer mental health and well-being as a result of the COVID-19 pandemic. However, it is further hypothesized that the methodologies used for studies will have an impact on how likely an effect is to be reported. Specifically, it is predicted that studies utilizing standardized assessments would be less likely to report finding any effect of COVID-19 on child mental health or well-being than those using researcher-made measures, as standardized measures are designed to be stable over time and are thus less sensitive to smaller or short-term change. It is further predicted that longitudinal studies would be less likely to report finding an effect than studies utilizing a cross-sectional study design, as longitudinal studies compare ratings for the same aspects of mental health or well-being across time and are less able to tap into areas of mental health or well-being that are uniquely affected by an event. Cross-sectional studies, on the other hand, may be developed in reaction to an event and can administer assessments based on commonly reported experiences, including already developed standardized measures, or newly created researcher-made measures. It is further hypothesized that there will be greater evidence for an impact in some more commonly researched areas of mental health (e.g., anxiety and depression) than for areas of mental health and well-being less frequently examined within child samples (e.g., post-traumatic stress disorder or positive affect). It is also expected that most relevant studies will focus exclusively on autistic children, rather than other SEND diagnoses. Currently there is a dearth of research comparing mental health or well-being outcomes across categories of SEND, so this review aims to highlight whether any clear differences exist between studies using different samples or whether the impact of COVID-19 has had a universal effect regardless of SEND category.

## Methods

The protocol for this review was developed a priori and published on OSF Registries (Registration https://doi.org/10.17605/OSF.IO/C7NEF), which includes further details about methodology choices.

A systematic review of relevant literature was initially conducted using five databases (ERIC, PubMed, PsycInfo, Scopus, and Web of Science). Each database was searched in January 2023 for titles, abstracts, and/or keywords corresponding to the following search terms: (COVID-19 OR coronavirus) AND (child*) AND ("special educational needs" OR "special needs" OR disability OR disabilities OR "neurodevelopmental disorder" OR "neurodevelopmental disorders" OR Autism OR "Down Syndrome" OR "William* Syndrome" OR "learning disabilities" OR "learning disability") AND ("psychosocial outcomes" OR "psychosocial outcome" OR "mental health" OR well-being OR wellbeing).

Additional searches were next conducted using OSF Registries (OSF preprints and PsyArXiv) and Google Scholar (first 150 results in original search, plus all 159 from 2022) to avoid missing relevant studies. Finally, a backwards citation search of earlier reviews on similar topics was conducted (Amaral & de Vries, 2020; Ameis et al., 2020; Bakkum et al., 2022; Baweja et al., 2021; Kreysa et al., 2022; Musa et al., 2021).

The inclusion/exclusion criteria for the review are displayed in Table 1. A range of methodologies were eligible to be included in the current view, so that no key findings were likely to be missed. However, it was a key requirement that the study must use empirical, replicable methods, to ensure the reliability of the evidence. Therefore, qualitative studies that did not explicitly set out to examine changes in mental health due to the COVID-19 pandemic were excluded, even if incidental findings were noted in the paper. It was also a key requirement that studies examined *change* in mental health/well-being; this meant that studies examining mental health/well-being during the COVID-19 pandemic without any comparison (either a retrospective measure or longitudinally) to previous mental health/well-being were excluded. SEND status was defined as having a disability or learning difference that makes it more challenging for the individual to learn using, or engage with, traditional education without additional support or adaptations. This included children who need additional support in school settings due to differing communication/interaction styles, those with cognitive or learning difficulties, those with long-term emotional or mental health difficulties, and those with sensory or physical needs. This definition was based on UK Department for Education guidance (Timpson, 2014). The authors understand that the chosen definition is broad and may differ from those preferred by different countries, and this needs to be considered when reflecting upon the findings of this review. By opting for a broad definition, and examining potential differences between SEND categories, it is hoped that important findings on groups considered to fall within the classification of SEND by some countries are less likely to be missed.

**Table 1:** Inclusion/Exclusion Criteria for the Review

Inclusion Criteria	Exclusion Criteria
Population: children (0—18 years) with Special Education Needs Disabilities	Comparing completely different participant samples at different time-points
Study must focus on impact of COVID-19	Treatment or intervention studies
Longitudinal or cross-sectional design	Studies without empirical data
Quantitative or qualitative measures	Conference papers or extended abstracts
Mental health or well-being (child) outcomes (standardised or researcher-made outcomes)	No access to full text in English

Reliability of the study was ensured by having four trained coders, two of which (VC and CJ) independently screened both the abstracts/titles and the full texts for searches conducted in 2020–21 and the other two (VS and DZ) for searches conducted in 2022. For the searches in 2020–21, at the abstract/title stage, a randomly assigned 10% of titles and abstracts (*n* = 87) were screened, while for the full-text stage, all papers (*n* = 228) were screened. Any discrepancies between the two coders were resolved through discussion, and OP and JVH made final decisions if needed. The inter-rater agreement for this first title/abstract screening stage was 92%, indicating strong or near-perfect agreement (K = 0.82). For the full-text stage, the inter-rater agreement was 90%, indicating substantial or moderate agreement (K = 0.74), as per McHugh (2012). This process led to the initial inclusion of 49 papers in the data extraction stage. For the searches in 2022, at the abstract/title stage, all titles, and abstracts (*n* = 739) were screened, and for the full-text stage, all papers (*n* = 119) were screened. Any discrepancies between the coders were resolved through a discussion, and VC made final decisions if needed. The inter-rater agreement for the title/abstract screening stage was 93%, indicating a moderate to substantial agreement (K = 0.73). For the full-text stage, the inter-rater agreement was 81%, indicating a moderate or substantial agreement (K = 0.62). This process led to the inclusion of an additional 18 papers in the data extraction stage.

To ensure consistency amongst the four coders, VC and VS screened a final sample of papers published in December 2022 to January 2023 at abstract/title stage (*n* = 103) and a sample of papers screened by each other at full-text (*n* = 104). Their inter-rater agreement for the title/abstract screening stage was 77% (K = 0.22) and for their full text agreement was 79% (K = 0.28). As the inter-rater reliability was poorer than expected, the reason for discrepancy was examined and it was determined that the discrepancies were due to cautious over-inclusiveness at both stages. Thus, it is unlikely that key texts had been missed during the screening process.

VC, VS, and DZ extracted the data, which included sample size and characteristics, methodology, and relevant findings. Some papers initially seemed to meet the inclusion criteria but were excluded from the review due to a lack of clear extractable results that directly focused on the impact of COVID-19 on the mental health and well-being of children with SEND. As recommended by PRISMA guidelines (Page et al., 2021), these papers are identified in the results section.

## Results

**Fig. 1:**
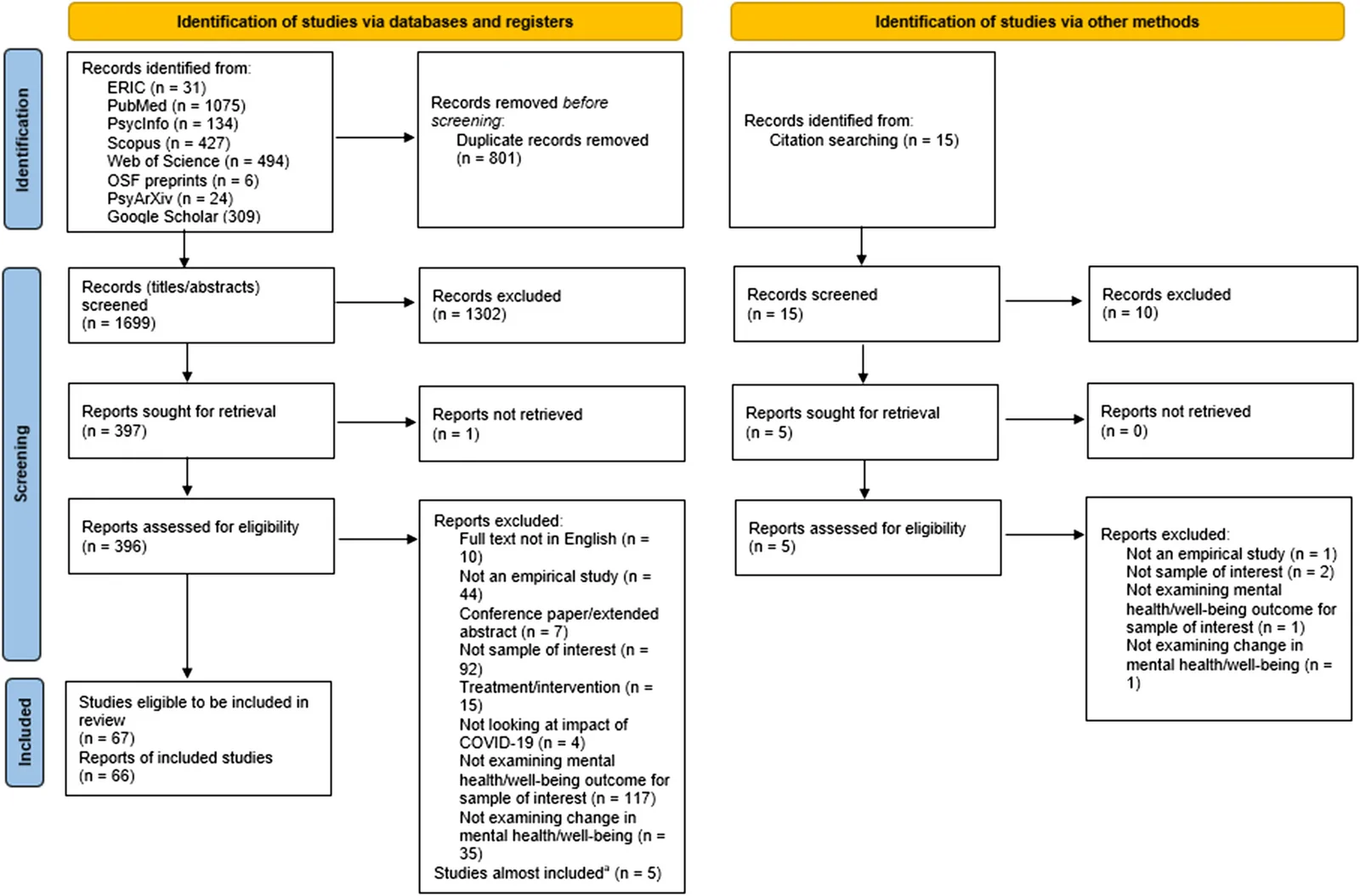
PRISMA Flowchart Showing Review Selection Process. ^a^ The studies almost included initially appeared to meet inclusion criteria, but were rejected at data extraction due to not fully meeting each criteria

Citations and abstracts returned from the search of the databases (including OSF Registries and Google Scholar searches; *n* = 2500) were imported into Mendeley (Reiswig, 2010). Duplicates (*n* = 801) were removed using both the ‘Check for Duplicates’ tool and by hand. The backwards citation search additionally found 15 further abstracts to review. Details about the number of papers identified from each source, the number of papers rejected at each stage, and reasons for rejection are presented in the PRISMA flowchart in Fig. 1. Additionally, five papers initially appeared to meet inclusion criteria, but were rejected from this review. Guller et al. (2021) initially seemed to meet criteria due to reference to the symptoms emerging during the pandemic, but after an examination of the materials used, this was not directly assessed in the study. Polónyiová et al. (2022a), Polónyiová et al. (2022b), and Paulauskaite et al. (2022) were excluded due to different participant samples being used at each time-point. Finally, Takahashi & Honda (2021) provided data about child well-being before and after restrictions came into effect, but only provided change statistics for the total sample and did not separate out the sub-set of children with neurodevelopmental disorders, thus was excluded despite initially appearing to meet inclusion criteria.

Sixty-seven studies were eligible for inclusion in this review. However, one study (Hosokawa et al., 2021) expanded on the findings of another (Kawabe et al., 2020), with all study and sample characteristics remaining the same. Only Hosokawa et al. (2021) is presented below; this did not lead to any study findings being missed, as relevant findings from Kawabe et al. (2020) are also presented in Hosokawa et al. (2021).

Out of the 66 studies included in this review, 61 examined change between pre-COVID-19 and post-COVID-19-onset, with the other 5 studies examining change over the pandemic period alone (Dvorsky et al., 2022; Raffagnato et al., 2021; Raw et al., 2021; Toseeb & Asbury, 2022; Waite et al., 2021). Out of the 61 studies examining change from pre-pandemic, the majority reported a deterioration of SEND child mental health/well-being for at least one reported analysis (n = 54; 89%). However, some studies reported different findings for different outcomes of interest. We therefore present the findings for each observed outcome separately. Furthermore, different studies used different approaches to determine whether a negative or positive impact on mental health/well-being could be established; the following results table thus clearly notes whether this was determined through traditional null hypothesis testing or descriptive comparisons (e.g., percentages of children affected or qualitative analysis). Twelve studies employed a qualitative design, fifty-one employed a quantitative design, and three used mixed methods.

Studies included in this review used samples of children from a variety of countries, with European countries being most common (12 from Italy, 11 from the UK, 4 from Spain, 1 from Portugal, 2 from France, 1 from Ireland, 1 from Belgium, 1 from the Netherlands, 1 from Sweden, and 1 from Poland), as well as North American countries (10 from the USA and 2 from Canada), but Asian countries were also reasonably represented (3 from Türkiye,^1^ 2 from Hong Kong, 1 from Malaysia, 1 from Japan, 1 from China, 1 from Bangladesh, 1 from Israel, and 1 from Saudi Arabia), as well as four studies conducted in Australia, one in Chile, and three in Africa (Malawi, Algeria, and South Africa).

Sample sizes varied from six to 6393, but the average reported sample size was 312 (SD = 839.12). Overall, gender ratios of the SEND samples reflected the greater number of boys with SEND diagnoses than girls (Hibel et al., 2010; Sullivan & Bal, 2013), with boys making up 66.1% of the samples in total. Most studies used a wide range of ages for the children included in their samples, with the overall range from birth to 18-years; the vast majority of studies (94%) included a broad range of ages, spanning at least three years. Most samples included autistic children, with a third solely using autistic samples and 45% using mixed samples. Other SEND categories reported as part of samples include ADHD, physical disabilities, learning disabilities, and genetic conditions (see Table 2 for full description).

**Table 2:** Study characteristics and reported impact of COVID-19 on mental well-being for children with SEND

Paper	SEND Category	Study Design	Methodology	Measure(s)	Reporter	Area of Mental Health/ Well-being	How Operationalized	Effect	How Established
Adams et al. (2022)	Autism	Cross-sectional	Qualitative	Interview	Parent/caregiver	Anxiety	Anxiety	Negative	Descriptive comparison
						Other	Stress	Negative	
Alenezi et al. (2022)	Mixed	Cross-sectional	Quantitative	Researcher-made	Parent/caregiver	Anxiety	Anxiety	Negative	Significance testing
Amorim et al. (2020)	Autism	Cross-sectional	Quantitative	Researcher-made	Parent/caregiver	Anxiety	Anxiety	Negative	Descriptive comparison
Arazi et al. (2022)	Autism	Cross-sectional	Quantitative	Researcher-made	Parent/caregiver	Anxiety	Anxiety	Negative	Significance testing
						Mood	Change in ‘general mood’	No change	
Asbury & Toseeb (2022)	Mixed	Repeated cross-sectional design	Qualitative	Open question on (researcher-made) questionnaire	Parent/caregiver	Anxiety	Anxiety	Negative	Descriptive comparison
						Other	‘Mental wellbeing’	Positive	
Asbury et al. (2021)	Mixed	Cross-sectional	Qualitative	Open question on (researcher-made) questionnaire	Parent/caregiver	Anxiety	Anxiety	Negative	Descriptive comparison
						Mood	Low mood	Negative	
						Other	Stress; distress	Negative	
Banerjee et al. (2021)	Mixed	Cross-sectional	Mixed	Researcher-made (closed and open questions)	Parent/caregiver	Anxiety	Anxiety	Negative	Descriptive comparison
						Other	Emotional well-being	Negative	
Berasategi Sancho et al. (2022)	Mixed	Cross-sectional	Quantitative	Researcher-made, but validated	Parent/caregiver	Anxiety	Nervous	Negative	Descriptive comparison
						Mood	More sad, crying more	Negative	
Bhat (2021)	Autism	Cross-sectional	Quantitative	Researcher-made	Parent/caregiver	Other	Emotional or mental health	Negative	Descriptive comparison
Bıyık et al. (2021)	Cerebral Palsy	Cross-sectional	Quantitative	Researcher-made, but validated	Parent/caregiver	Anxiety	**Anxiety**/stress	Negative	Descriptive comparison
						Other	Anxiety/**stress**	Negative	
Bobo et al. (2022)	Autism	Cross-sectional	Quantitative	Researcher-made	Parent/caregiver	Other	Emotional well-being	Negative	Descriptive comparison
Boterberg et al. (2022)	Autism	Cross-sectional	Quantitative	Researcher-made	Parent/caregiver	Anxiety	‘Anxious or down’	Negative	Significance testing
						Mood	‘Anxious or down’	Negative	
Cacioppo et al. (2021)	Physical disabilities	Cross-sectional	Quantitative	Researcher-made, but piloted	Parent/caregiver	Mood	Morale	Negative	Descriptive comparison
						Other	Eating disorders	Negative	
Charalampopoulou et al. (2022)	Autism	Cross-sectional	Quantitative	Researcher-made	Parent/caregiver	Other	Mental health	Negative	Descriptive comparison
Conti et al. (2020)	Mixed	Longitudinal	Quantitative	Standardized: Child Behaviour Check List (Achenbach & Rescorla, 2001)	Parent/caregiver	Anxiety	Anxiety	Negative	Significance testing
						Mood	Withdrawn/depressed	No change	
						Other	Obsessive–Compulsive symptoms, Post-Traumatic Stress symptoms	Negative	
Corbett et al. (2021)	Autism	Longitudinal	Quantitative	Standardized: State–Trait Anxiety Inventory for Children (STAI-C; Spielberger, 1973); Responses to Stress Questionnaire (RSQ; Connor-Smith et al., 2000)	Child	Anxiety	Anxiety	No change No change	Descriptive comparison
						Other	Stress		
Cost et al. (2022)	Mixed	Cross-sectional	Quantitative	Adapted version of measure developed during pandemic for another study: the international CRISIS Questionnaire (Nikolaidis et al., 2021)	Parent/caregiver and child	Anxiety	Anxiety	Negative	Descriptive comparison
						Mood	Depression	Negative	
						Other	Obsessive–Compulsive symptoms	Negative	
De Giacomo et al. (2021)	Mixed	Longitudinal	Quantitative	Standardized: Child Behaviour Check List (Achenbach & Rescorla, 2001)	Parent/caregiver	Other	Emotional/behavioural problems	No change	Significance testing
Di Giorgio et al. (2021)	Fragile-X syndrome	Cross-sectional (but treated as if longitudinal)	Quantitative	Standardized: Child Adjustment and Parent Efficacy Scale-Developmental Disability (Mazzucchelli et al., 2011)	Parent/caregiver	Other	Emotional problems	No change	Significance testing
Dondi et al. (2021)	Learning disabilities	Cross-sectional	Quantitative	Researcher-made	Parent/caregiver	Mood	Mood	Negative	Descriptive comparison
Dvorsky et al. (2022)	Attention Deficit Hyperactivity Disorder	Longitudinal (but post-lockdown only)	Quantitative	Adapted version of measure developed during pandemic for another study: the international CRISIS Questionnaire (Nikolaidis et al., 2021)	Child	Other	Stress; mental health	Post-lockdown only	Significance testing
Elphick et al. (2022)	Mixed	Cross-sectional	Quantitative	Researcher-made	Parents/caregiver	Anxiety	Anxiety	Negative	Descriptive comparison
						Mood	Mood	Mixed	
Fong et al. (2021)	Autism	Cross-sectional (but treated as if longitudinal)	Quantitative	Standardized: The Conners' Parent Rating Scales-3 (Conners, 2015)	Parent/caregiver	Anxiety	Anxiety	Negative	Significance testing
						Mood	Depression	Negative	
						Other	Stress	Negative	
Fridell et al. (2022)	Mixed	Cross-sectional	Qualitative	Researcher-made	Parent/caregiver and child	Anxiety	Worry/anxiety	Negative	Descriptive comparison
						Mood	Sense of meaningless/poor motivation	Negative	
						Other	General well-being/sense of thriving	Positive	
Fumagalli et al. (2021)	Autism	Cross-sectional	Quantitative	Researcher-made	Parent/caregiver	Mood	Mood changes	Negative	Descriptive comparison
Genova et al. (2021)	Autism	Cross-Sectional	Quantitative	Standardized: Covid-19 Adolescent Symptom and Psychological Experience Questionnaire (CASPE; Ladouceur, 2020)	Parent/caregiver	Other	Meltdowns; emotional change	Negative; no change	Descriptive comparison
Graziola et al. (2020)	Tourette's syndrome and chronic tic disorders	Cross-sectional	Quantitative	Researcher-made (anxiety and mood); standardized (OCD only): Children's Yale–Brown Obsessive–Compulsive Scale (CY‐BOCS; Bejerot et al., 2014)	Parent/caregiver and child	Anxiety	Anxiety	Negative	Descriptive Comparison and Significance Testing (OCD severity only)
						Mood	Mood	Negative	
						Other	Obsessive–Compulsive symptoms	Mixed	
Hannawi et al. (2022)	Autism	Cross-Sectional	Quantitative	Researcher-made	Parent/caregiver	Anxiety	Anxiety	No change	Descriptive comparison
						Other	Stress	Negative	
Hornstra et al. (2022)	Mixed	Cross-Sectional (but treated as if longitudinal)	Quantitative	Researcher-made	Parent/caregiver	Mood	Motivation	Mixed	Significance testing
						Other	Well-being	Negative	
Hosokawa et al. (2021)	Autism	Cross-sectional	Quantitative	Researcher-made	Parent/caregiver	Other	Stress	Negative	Descriptive comparison
Houghton et al. (2022)	Mixed	Longitudinal	Quantitative	Standardized: Children’s Depression Inventory-2 (Kovaks, 2004); The Warwick-Edinburgh Mental Wellbeing Scale (Tennant et al., 2007); The Strengths and Difficulties Questionnaire (Goodman, 1997)	Child	Anxiety	Internalizing symptoms	No change No change	Significance testing
						Mood	Depression; internalizing symptoms	No change	
						Other	Positive well-being		
Jordan et al. (2022)	Fragile-X syndrome	Cross-sectional	Qualitative	Open question on (researcher-made) questionnaire	Parent/caregiver	Anxiety	Anxiety	No change	Descriptive comparison
						Mood	Depressed mood	Negative	
Kaczynski et al. (2021)	Chronic pain disorder	Cross-Sectional (but treated as if longitudinal)	Quantitative	Standardized: The Patient Reported Outcomes Measurement Information System—Short Form (PROMIS; Cella et al., 2010)	Parents/caregiver and child	Anxiety	Anxiety	No change	Significance testing
						Mood	Depression	No change	
Layachi & Schuelka, (2022)	Mixed	Cross-sectional	Qualitative	Researcher made	Parent/caregiver and child	Mood	Sadness	Negative	Descriptive comparison
						Other	Emotional distress; stress	Negative	
Lew-Koralewicz (2022)	Autism	Cross-sectional	Qualitative	Open question (researcher-made) questionnaire	Child	Anxiety	Anxiety	Negative	Descriptive comparison
						Mood	Sadness	Negative	
						Other	Emotional problems; general mental health/well-being	Negative; positive	
Lopez-Serrano et al. (2021)	Mixed	Cross-sectional	Quantitative	Researcher-made	Parent/caregiver	Anxiety	Anxiety/restlessness	Negative	Descriptive comparison
						Mood	Sadness/discouragement	Negative	
						Other	Eating disorder symptoms; Obsessive–Compulsive symptoms; agoraphobia	No change	
Lugo-Marín et al. (2021)	Autism	Longitudinal	Quantitative	Standardized: Child Behaviour Check List (Achenbach & Rescorla, 2001)	Parent/caregiver	Anxiety	Anxiety/ depression/withdrawal	No change	Significance testing
						Mood	Anxiety/ depression/ withdrawal	No change	
Masi et al. (2021)	Mixed	Cross-sectional	Quantitative	Researcher-made, but pilot tested	Parent/caregiver	Anxiety	Anxiety disorder symptoms	Negative	Descriptive comparison
						Other	Stress	Negative	
Mete Yesil et al. (2022)	Mixed	Cross-sectional	Quantitative	Researcher-made	Parent/caregiver	Mood	Crying attacks	Negative	Descriptive comparison
Montirosso et al. (2021)	Mixed	Cross-sectional	Quantitative	Standardized: modified Child Behaviour Check List—PTSD scale (Dehon & Scheeringa, 2006)	Parent/caregiver	Anxiety	Anxious/ depressed	Negative	Descriptive comparison
						Mood	Anxious/ depressed	Negative	
Morgül et al. (2022)	Mixed	Cross-sectional	Quantitative	Standardized: Strengths and Difficulties Questionnaire – parent report (SDQ; Goodman, 2001)	Parent/caregiver	Anxiety	Anxiety and worry	Negative	Descriptive comparison
						Mood	Sad	Negative	
O’Sullivan et al. (2021)	Autism	Cross-sectional	Qualitative	Semi-structured interview	Parents and SEND teachers	Anxiety	**Anxiety**/stress	Negative	Descriptive comparison
						Other	Anxiety/**stress**	Negative	
Operto et al. (2022)	Mixed	Longitudinal	Quantitative	Standardized: Child Behaviour Check List (Achenbach & Rescorla, 2001)	Parent/caregiver	Anxiety	Anxiety/depression; anxiety problems	Negative	Significance testing
						Mood	Anxiety/depression; withdrawal/depression	Negative	
Ozsivadjian et al. (2022)	Autism	Cross-Sectional	Qualitative	Researcher-made	Parent/caregiver	Anxiety	Anxiety	Mixed	Descriptive comparison
						Mood	Depression; lack of motivation	Negative	
						Other	Meltdowns	Negative	
Panjwani et al. (2021)	Autism	Cross-sectional	Quantitative	Researcher-made	Parent/caregiver	Mood	Crying	Negative	Descriptive comparison
						Other	Obsessive–Compulsive symptoms	Negative	
Pastori et al. (2021)	Mixed (disability other than learning disability)	Cross-sectional	Quantitative	Researcher-made	Parent/caregiver	Mood	Mood changes	Negative	Descriptive comparison
Paulauskaite et al. (2021)	Developmental Delay	Cross-sectional	Mixed	Researcher-made	Parent/caregiver	Other	Mental health; stress	Negative	Descriptive comparison
Pellicano et al. (2022)	Autism	Cross-sectional	Qualitative	Semi-structured interviews	Child	Mood	Sadness	Negative	Descriptive comparison
						Other	Obsessive–Compulsive symptoms; stress	Negative	
Rabbani et al. (2021)	Autism	Longitudinal	Quantitative	Researcher-made	Parent/caregiver	Mood	Mood swings	No change	Significance testing
Raffagnato et al. (2021)	Mixed	Longitudinal	Quantitative	Standardized: Child Behaviour Check List and Youth Self Report (Achenbach & Rescorla, 2001)	Parent/caregiver and child	Anxiety	Internalizing	Post-lockdown only	Significance testing
						Mood	Internalizing	Post-lockdown only	
						Other	PTSD	Post-lockdown only	
Raw et al. (2021)	Mixed	Longitudinal	Quantitative	Standardized: Strengths and Difficulties Questionnaire – parent report (SDQ; Goodman, 2001)	Parent/caregiver	Other	Emotional problems	Post-lockdown only	Significance testing
Rizzo et al. (2021)	Mixed	Cross-sectional	Quantitative	Researcher-made	Parent/caregiver	Anxiety	Anxiety disorder symptoms	Negative	Descriptive comparison
						Other	Obsessive–Compulsive symptoms; stress; negative emotions	Negative	
Sakız (2021)	Physical disabilities	Cross-sectional	Qualitative	Structured interview	Child	Anxiety	Anxiety/fear	Negative	Descriptive comparison
						Mood	Hopelessness	Negative	
Santa-Cruz et al. (2022)	Mixed	Longitudinal	Quantitative	Standardized: Child Behaviour Check List (Achenbach & Rescorla, 2001)	Parent/caregiver	Anxiety	Internalizing	Mixed	Significance testing
						Mood	Internalizing	Mixed	
Sciberras et al. (2022)	Attention Deficit Hyperactivity Disorder	Cross-Sectional (but treated as if longitudinal)	Quantitative	Researcher-made	Parent/caregiver	Mood	Sad/depressed	Negative	Significance testing
Segre et al. (2021)	Mixed	Cross-sectional	Quantitative	Standardized: adaption of the Short Mood and Feeling questionnaire (Angold et al., 1995)	Child	Mood	Mood	Negative	Descriptive comparison
Singal et al. (2021)	Mixed	Cross-sectional	Quantitative	Researcher-made	Parent/caregiver	Anxiety	Anxious	Negative	Descriptive comparison
						Mood	Sad	Negative	
Soriano-Ferrer et al. (2021)	Dyslexia	Longitudinal	Quantitative	Standardized: STAI-C (Spielberger, 1973); Children’s Depression Inventory-Short Form (Kovacs, 1992); the Spanish version of the Strengths and Difficulties Questionnaire (SDQ) (Goodman, 2001; Ortuño-Sierra et al., 2017)	Parent and child	Anxiety	State anxiety	Negative	Descriptive comparison
						Mood	Depression	Negative	
						Other	Emotional symptoms	Negative	
Su et al. (2021)	Mixed	Cross-sectional (but treated as if longitudinal)	Quantitative	Researcher-made (part of larger study)	Parent/caregiver	Anxiety	Anxiety	Negative	Significance testing
Sutter et al. (2021)	Motor disability	Cross-sectional	Mixed	Researcher-made	Parent/caregiver	Anxiety	Stress/**anxiety**	Negative	Descriptive comparison
						Mood	Depression	Negative	
						Other	**Stress**/anxiety; mental health	Negative	
Theis et al. (2021)	Mixed	Cross-sectional	Quantitative	Researcher-made and also adapted standardized measure: adapted Strength and Difficulties Questionnaire (Goodman, 1997)	Parent/caregiver	Anxiety	Anxiety	Negative	Descriptive comparison
						Mood	Mood	Negative	
						Other	Mental health	Negative	
Toseeb & Asbury (2022)	Mixed	Longitudinal	Quantitative	Standardized: the anxiety scale for children with autism spectrum disorder (Rodgers et al., 2016); the low mood subscale of the Revised Child Depression and Anxiety Scale (Chorpita et al., 2000)	Parent/caregiver	Anxiety	Anxiety	Post-lockdown only	Significance testing
						Mood	Depression	Post-lockdown only	
Vasa et al. (2021)	Autism	Cross-sectional	Quantitative	Researcher-made	Parent/caregiver	Anxiety	Anxiety disorder symptoms	Negative	Descriptive comparison
						Mood	Depression	Negative	
						Other	Psychiatric symptoms; Obsessive–Compulsive symptoms	Negative	
Waite et al. (2021)	Mixed	Longitudinal	Quantitative	Standardized: Strengths and Difficulties Questionnaire – parent report (SDQ; Goodman, 2001)	Parent/caregiver	Other	Emotional symptoms	Post-lockdown only	Significance testing
Wong et al. (2021)	Autism or other developmental disorders/delays	Cross-sectional	Qualitative	Focus group	Social work and psychological professionals	Other	Emotional problems; positive affect	Negative; positive	Descriptive comparison
Yarger et al. (2021)	Autism	Longitudinal	Quantitative	Standardized: the Screen for Child Anxiety Related Disorders (Birmaher et al., 1997); Child Behaviour Check List (Achenbach & Rescorla, 2001)	Parent/caregiver	Anxiety	Anxiety	No change	Significance testing
						Mood	Depression	Positive	

NB: Study details refer to analyses of interest, and thus cross-sectional results within a longitudinal study will be referred to as cross-sectional; ‘Other’ refers to areas of mental health/well-being other than the most commonly examined areas of anxiety and mood

### Reported Impact on Mental Health and Well-Being

A range of mental health and well-being outcomes were explored across the included studies (see Table 2). As several of the included studies utilized samples with young children, it was common for respondents to be asked to report on the child’s change in symptoms of ‘worry’ or ‘low mood’, or even to assess changes in frequency of crying as a means of tapping into the constructs, rather than referring to clinical terms such as anxiety or depressive symptoms. As studies asking respondents about worry or anxiety, for example, were attempting to tap into the same construct, it seemed logical to group studies together on the basis of construct so that we could determine whether COVID-19 had a greater effect on one aspect of mental health or well-being over another. Where studies presented combined findings for anxiety and depression (including when operationalizing as internalizing symptoms), findings are presented for both summaries.

Most of the included studies examined anxiety (including ‘worry’ or ‘nervousness’) as an outcome measure (*n* = 42); two of these studies only explored the trajectory of anxiety levels from early lockdown to the return of face-to-face education (Raffagnato et al., 2021; Toseeb & Asbury, 2022). Of the 40 studies that compared anxiety levels during the pandemic to previous levels, 32 (80%) reported an overall negative impact, with anxiety levels increasing from before to during the pandemic. For example, qualitative research highlighted that the closure of schools, change in routine, fear of death of loved ones, and the thought of vaccinations caused anxiety to children with SEND (e.g. Adams et al., 2022; O’Sullivan et al., 2021; Sakız, 2021). One study used a qualitative design to assess whether parents reported greater anxiety in early lockdown compared to later in the course of the pandemic (Asbury & Toseeb, 2022), finding that the impact of the COVID-19 pandemic on child anxiety was greatest in early lockdown, before slowly decreasing and then increasing as children re-entered face-to-face education. Notably, Sakız (2021) reported that online learning alternatives to traditional education were not as effective for protecting children against high anxiety, as children lacked both the physical health (via sports and active lessons) and social benefits that traditional face-to-face education provides.

Quantitative studies found that children were frequently considered to be more anxious than they were previously (e.g., Berasategi Sancho et al., 2022; Fong et al., 2021). Alenezi and colleagues (2022) in particular provided evidence with their cross-sectional study that anxiety increased from pre-pandemic levels during the early pandemic, and then increased again later in the pandemic. Five studies found that anxiety was typically negatively impacted, but in some cases this impact was not seen. For example, Bıyık and colleagues (2021) found that, for their sample of children with cerebral palsy, anxiety and stress increased for every four in ten children, but decreased for one in ten. Equally, Toseeb & Asbury (2022) reported that initially high anxiety levels decreased over the course of lockdown for most children with SEND, but for autistic children there was no significant change in anxiety over time. These findings suggest that there may have been a heterogenous response to the pandemic in terms of anxiety levels. This suggestion is further supported by evidence from four studies that did not find a change in anxiety levels from before to after the start of the pandemic (e.g., Corbett et al., 2021). Interestingly, and in opposition to the findings of Toseeb & Asbury (2022), Yarger and colleagues (2021) found that although there was no significant change in anxiety symptoms from pre- to post-lockdown, there was a decrease in anxiety symptoms over the post-lockdown time-points for autistic children. Although Banerjee et al. (2021) did note that one parent reported their child as being less anxious than typical during the lockdown, around two thirds of parents (with a sample of 53) reported that their children were more anxious. Furthermore, Ozsivadjian et al. (2022) found that for half of their autistic sample, anxiety actually decreased; however, anxiety did increase for some. It is important to note that although there was some variation in the impact on anxiety levels for children with SEND, studies typically reported a negative impact.

Just as with anxiety, impact on child depressive symptoms/mood was examined for most of the included studies (*n* = 41); again, two studies examined post-pandemic change only. For these studies, changes in mood were operationalised as low mood, depressive symptoms, mood swings, sadness, frequency of crying, and feelings of hopelessness. Again, most studies examining change from before to after the pandemic start reported a generally negative impact of the pandemic on mood (*n* = 30; 73%), with children noted as crying more frequently (Berasategi Sancho et al., 2022; Panjwani et al., 2021) and experiencing greater symptoms of depression (e.g., Cost et al., 2022; Fong et al., 2021; Soriano-Ferrer et al., 2021). However, several studies did note that this was not the case for all children with SEND, with one study reporting that depressed mood only increased for 6.5% of the sample of children with Fragile-X Syndrome (Jordan et al., 2022), despite parents commenting that this group struggled with the lack of routine. Furthermore, Cacioppo et al. (2021) reported that morale was negatively impacted for a little under half of the children with physical disabilities (43%), but positively impacted for a smaller proportion of children (13%). Similarly, autistic children in Fumagalli et al.’s (2021) study reported greater negative mood changes than positive, yet were more likely to report positive mood changes than the typically developing (TD) control group. A small number of studies reported no change in mood symptoms (*n* = 8), with Conti et al. (2020) finding no difference over time in symptoms of depression and withdrawal for older children in their sample. Toseeb & Asbury (2022) found that over the first six months of lockdown, depression symptoms did not change over time for their UK-based SEND sample. One further study found that for children with autism, depressive symptoms actually decreased between the pre-COVID-19 and post-lockdown time-points and then stayed stable throughout the pandemic time-points (Yarger et al., 2021).

Although most included papers examined the impact of the COVID-19 pandemic on child anxiety or depression/mood, other mental health and well-being outcomes were also explored. A number of studies referred more generally to attempting to examine changes in mental health, psychological distress, emotional well-being, or negative emotions (*n* = 21), with just over half (*n* = 12) reporting a clear deterioration of mental health/well-being for at least one measure. Paulauskaite et al. (2021) stated that a large proportion of parents reported having to manage additional mental health needs during the pandemic for their child with developmental delay, with poorer mental health claimed by one parent to be due to a lack of specialist support and outdoor activity opportunities. Vasa and colleagues (2021) reported that child psychiatric problems were more likely to be reported when a family member had contracted COVID-19. Genova et al. (2021) reported that whilst there was no substantial change in negative or positive emotions for the majority of their autistic sample, there was an increase in meltdowns experienced. Dvorsky et al. (2022) noted that for their sample of adolescents with ADHD, the impact of the pandemic on their mental health stayed stable over the course of the pandemic. Finally, Bhat (2021) noted that although mental health was negatively impacted for most SEND children, children who understood less about the pandemic or had a greater language delay were considered to be less affected by it, and children with greater repetitive behaviors were considered to be more negatively affected. These findings may suggest that individual differences between children may have led to different experiences of the pandemic. Alternatively, it may be that parents whose children are not able to communicate their distress verbally are less likely to understand the full extent of their child’s distress. However, Asbury & Toseeb (2022) did also find that for a minority of autistic children, their mental well-being increased whilst education was delivered remotely, due to the previous negative effects of the school environment on their mental health, with their mental well-being decreasing once face-to-face education restarted. Similarly, Wong et al. (2021) had noted that some children with autism or developmental delay had experienced greater positive affect due to no longer experiencing bullying during school, and Banerjee et al. (2021) equally noted that for at least one child an improvement in mental health due to not needing to attend school face-to-face helped with concentration with school work. Finally, four studies exploring the impact of the pandemic on emotional problems found no evidence of a change in problems, and another two studies, utilizing an overlapping dataset with one another, found that over the course of the pandemic emotional symptoms decreased (Raw et al., 2021; Waite et al., 2021).

Stress was also commonly examined (*n* = 10), with most studies presenting findings of increased stress for their samples. Dvorsky et al. (2022) additionally found that the impact on stress levels may have been greater earlier in the pandemic. Stress may have been elicited by the social restrictions that were imposed during this time (Paulauskaite et al., 2021; Rizzo et al., 2021). For Hosokawa et al.’s (2021) autistic sample, the main reported stress factors were being refrained from playing outside, then change in daily routine, being restricted from seeing their friends and family, and finally not having face-to-face education.

Research also noted that children may have experienced an exacerbation of previous mental health difficulties, such as symptoms of post-traumatic stress disorder (PTSD; Conti et al., 2020), or developed novel mental health difficulties, such as eating disorders (Cacioppo et al., 2021; Graziola et al., 2020; Sutter et al., 2021). However, Lopez-Serrano et al. (2021) found that symptoms of eating disorders did not change substantially during the pandemic. Raffagnato et al. (2021) further found a decrease in PTSD symptoms between lockdown restrictions during the early pandemic and 4-months-later. Mixed findings were seen for studies examining the impact of the pandemic on symptoms of obsessive–compulsive disorder (OCD), with Panjwani et al. (2021) noting an increase in compulsive behaviors and obsessive thoughts for just under half and around a third of the sample respectively. Lew-Koralewicz (2022) No significant difference in OCD severity was reported for Graziola and colleagues (2020); however, there was a significant increase in compulsive behaviors and contamination obsessions specifically (although surprisingly not cleaning compulsions).

Notably, research greatly focused on examining mental health outcomes, rather than exploring change in positive well-being. However, as noted above, a number of studies did report positive changes for some children. For example, Fridell et al. (2022) reported that some autistic youth had felt like they were thriving during the pandemic. For a small number of studies (*n* = 5) a clear positive effect was seen for the sample. Additionally, for many of the studies reporting an overall negative effect, a mixed effect, or no overall significant effect, a proportion of the child samples were noted as having had decreases in mental health problems upon the start of the pandemic. For example, Bıyık et al. (2021) found that although around 40% of children experienced an increase in stress and anxiety, roughly 10% of the sample displayed a decrease in these symptoms. Additionally, Yarger and colleagues (2021) documented a decrease in depressive symptoms for autistic children upon commencement of lockdown restrictions. Similarly, Fumagalli and colleagues (2021) found that some autistic children under the age of six became calmer during lockdown. Other studies found that specific sub-groups within the sample may have been impacted differently to one another (e.g. Hornstra et al., 2022; Santa-Cruz et al., 2022).

Despite the majority of studies finding evidence for a negative impact of the COVID-19 pandemic on child mental health or well-being, there was variation within samples, even for studies reporting a negative impact. For example, for Banerjee et al.’s (2021) sample, although 79% of parents reported that their child’s emotional well-being had been affected, no parents rated the extent of the impact to be extreme. Some studies provided evidence of factors such as parental mental health (Bhat, 2021; Su et al., 2021; Vasa et al., 2021; Yarger et al., 2021) and household income (Bhat, 2021; Santa-Cruz et al., 2022; Vasa et al., 2021; Yarger et al., 2021) playing a role in the likelihood of children experiencing poorer mental health over the pandemic. Equally, some studies reported that parents were concerned about the lack of support available for their child (Bhat, 2021; Theis et al., 2021), and that level of available support was associated with child mental health outcomes (Sutter et al., 2021; Theis et al., 2021). The extent to which a routine could be maintained was also a factor in the extent to which some children experienced distress during the pandemic (Amorim et al., 2020; Hosokawa et al., 2021; Jordan et al., 2022).

### Role of Methodology on Reported Findings

The role of different methodological characteristics was examined for studies comparing pre- and post-COVID-19 outcomes. Findings were considered to be mixed if the impact depended either on which exact outcome was being examined or if at least one sub-set of the sample did not experience a negative impact for the observed outcome.

Eleven studies employed a qualitative design, with all of these studies finding some evidence of a negative impact on child mental health/well-being, but more than half (55%) of these studies reported mixed results. Fifty studies employed a quantitative methodology, with most of these finding evidence of some negative impact (*n* = 42; 84%); thirteen of these studies (31%) found mixed results. Three mixed method designs were utilized, all reporting a predominately negative impact.

Within the papers utilizing a quantitative (or mixed methods) approach, most used a cross-sectional design (*n* = 34), with 26% of these studies finding mixed results and the rest reporting only a negative impact (74%). There were six further cross-sectional studies that collected data at one time-point, but analysed the data as if longitudinal, with respondents asked to reflect back on the child’s mental health symptoms from before the start of the pandemic; evidence for some negative impact was seen for four of these studies, with one of these reporting mixed findings. Longitudinal studies (studies where data has been collected over several time-points), on the other hand, were less likely to find an effect. Out of the ten studies utilizing longitudinal designs, four found a negative impact for children with SEND (40%), with half of these findings mixed for analyses.

Thirty-three out of thirty-four studies utilizing researcher-made quantitative measures reported some negative impact (97%), compared to eleven out of eighteen using standardized measures (61%). Solely negative effects were reported for 76% of studies using researcher-made measures and 33% of studies using standardized measures.

Around twice as many studies using descriptive comparisons to compare changes in child mental health or well-being from pre- to post-COVID-19 onset reported a negative impact compared to studies employing significance testing methods (37.5% vs. 76.1% respectively).

In terms of the respondent, the majority of studies asked parents or other adults (e.g. teachers) to report on the child’s mental health (*n* = 48; 79%), but a few asked the young person to report on their own anxiety (*n* = 13; 21%), sometimes in addition to the parent or alternative adult respondent. When youth were asked to report on their own anxiety, a negative impact was typically reported (77%), with most of these reporting consistent negative effects (54% of all youth-reported studies). This was, however, less frequent than when using parent only reports (92% for some evidence of a negative effect, including 58% with consistent negative effects).

There was no clear difference for different categories of SEND. When comparing study findings on anxiety for different SEND categories, a negative impact of COVID-19 is seen across categories, especially when taking into account that many of the studies used mixed SEND samples (*n* = 22). Interestingly, Vasa and colleagues (2021) found that anxiety disorders were the psychiatric diagnoses most frequently reported as a newly arising mental health diagnosis during the pandemic for their autistic sample. Comparably to the findings for anxiety, a negative impact was found for mood for most studies, regardless of SEND categories. It should be noted that Hornstra et al. (2022) did find a difference in change in motivation levels for children based on SEND category, with children with specific learning difficulties being more adversely affected than autistic children or those with ADHD. Additionally, child characteristics such as greater repetitive behaviors, difficulties engaging in social communication, and greater motor impairment were also all linked to poorer mental well-being in Bhat’s (2021) sample. Alternatively, a poorer understanding of COVID-19 and its implications may have acted as a buffer against mental health problems for some children (Bhat, 2021; Vasa et al., 2021). Toseeb & Asbury (2022) further reported higher depression symptoms for children in mainstream (compared to specialist SEN) schools.

Overall, studies comparing SEND and non-SEND groups did not provide clear evidence for a greater effect of the pandemic on children with SEND. When studies used control groups to determine whether similar effects were seen for SEND and TD groups, some studies reported that similar patterns could be seen (Corbett et al., 2021; Fong et al., 2021; Fumagalli et al., 2021; Hosokawa et al., 2021; Segre et al., 2021) and others noted that different effects could be seen for SEND groups, with a greater impact for children with SEND on emotional well-being and mood (Berasategi Sancho et al., 2022; Cost et al., 2022; Dondi et al., 2021). A couple of papers noted that autistic sub-groups experienced poorer mental health, especially if there was a prior mental health diagnosis (Cost et al., 2022; Toseeb & Asbury, 2022). Dvorsky et al. (2022) further found that the group of adolescents with ADHD were more adversely affected than the TD group later in the pandemic, but similarly affected earlier. Furthermore, Waite and colleagues (2021) found that the sub-group of SEND children was more greatly impacted by the pandemic than the TD control group, but only if the children were younger (aged 4 to 10-years-old, compared to 11 to 16-years-old). Studies did, however, provide clearer evidence for poorer baseline mental health for SEND samples (e.g. Vasa et al., 2021; Yarger et al., 2021).

## Discussion

This systematic review set out to examine the impact of the COVID-19 pandemic on the mental health and well-being of children with SEND based on the available literature. In total, 66 studies met the inclusion criteria and were included in the current review. Studies were varied in their methodological approach, sample demographics, sample size, country of data collection, and gender ratio. Overall, studies tended to use larger samples of boys than girls and tended to employ a quantitative, cross-sectional approach to data collection.

### Impact of COVID-19 on the Mental Health/Well-Being of Children with SEND

The literature on the whole suggested a negative impact of the COVID-19 pandemic on the mental health and well-being of children with SEND, with the greatest evidence being presented for a negative effect on anxiety and mood, as hypothesized. However, there was evidence across the literature of individual variability, with some children affected more negatively than others, and even some cases of improvements in particular forms of mental well-being. It’s important to remember how much distress can be caused for this group by typical school and societal expectations outside of a pandemic (e.g., Adams et al., 2018). For some children the negative effects that may be imposed by structured education or experiencing bullying at school were alleviated, but not for all. Although more studies reported negative changes in mental health or well-being than positive changes, it is notable that many studies only asked respondents about possible negative impacts and that for several studies a clear positive effect was reported. These studies revealed that remote education provided potential for greater concentration on schoolwork (Banerjee et al., 2021), reduced incidence of bullying (Wong et al., 2021), and reduced exposure to energy-draining sensory triggers normally experienced in the classroom (Fridell et al., 2022), which had knock-on effects on mental health and positive well-being. Other studies noted a more general positive impact as a result of the child not being required to attend school in person during lockdown periods (Asbury & Toseeb, 2022; Yarger et al., 2021). Asbury & Toseeb (2022) reported that child anxiety linked to school attendance reduced for some children during lockdown, only to increase again once face-to-face schooling returned. Despite these experiences of temporarily improved mental health not being universal for SEND children during the pandemic, what is clear is that greater support is needed for children who may ordinarily be placed at higher risk of mental health problems and poorer well-being by traditional methods of education delivery (i.e. face-to-face schooling). It is possible that one reason for the greater number of studies conducted into the impact of COVID-19 for autistic children than for children with ADHD or specific learning difficulties, is that researchers expected there to be a more negative impact for autistic children; perhaps for students with ADHD researchers more readily acknowledged the possibility that a change to traditional education may not be considered negative by all students. More research on the potential benefits of remote learning on a long-term basis for students who find traditional face-to-face education challenging, as well as efforts to reduce causes of distress (e.g. bullying behaviour and noise levels in the classroom), is much needed.

The findings from the current review suggest that there was great variation in the experiences of SEND children during the COVID-19 pandemic. These individual differences for children with SEND in the studies included in the current review mirror the individual experiences that children with autism faced during school transitions during the COVID-19 pandemic (Code et al., 2022), with variation seen between children as to how they experienced this change during the pandemic due to variation in the type of support required by children. Similarly, within the literature presented in this review, some parents and children reported perceiving having less support during this time as well as a removal of services that were previously utilised (e.g., Theis et al., 2021), but this reduction in support services likely affected some groups more than others depending on country and region of residence, category of SEND, child age, and whether services were accessed prior to the pandemic start. Other research has indicated that loss of support for children and families during the pandemic was a major cause of stress for families (O’Hagan & Kingdom, 2020). Another study examining types of support required by families of children with SEND reported that families greatly differed in how adequate they felt the support they had received during the pandemic had been (Toseeb et al., 2020).

### Research Findings for Different Categories of SEND

Although the majority of studies included in this review used samples including autistic children, research has painted a similar picture for different groups of children with SEND, with evidence of a typically negative effect of the COVID-19 pandemic that is universal across SEND categories. However, it is possible that precise child characteristics (e.g. social communication differences and extent of understanding about COVID-19) were more predictive of child mental health outcomes than broader SEND categories.

Studies comparing SEND and non-SEND groups typically found similar trajectories for groups, yet a few studies did report that SEND groups were more adversely affected. This difference in effects may be partly due to methodology, with Dvorsky et al. (2022) finding in their longitudinal study that this differed depending on when the groups were compared. Perhaps groups of children with SEND may have had a more lasting negative impact of the pandemic than TD children, who were more easily able to settle back into normal life after restrictions eased. This is likely to be particularly the case for children who are at greater risk of serious illness if they contract COVID-19. Interestingly, Raw et al. (2021) and Waite et al. (2021) alternatively found that emotional problems for children with SEND actually decreased more over time, yet this was likely due to the particularly high reported emotional problems at baseline for these children. Similarly, Yarger et al. (2021) found that although the TD group experienced an increase in depressive symptoms, the autistic group showed the opposite effect over time. It must be noted, however, that children completed measures at different times to one another, making detailed understanding of the different trajectories more difficult to elicit here. In the wider literature, Bosch et al. (2022) noted that their SEND sample were also less adversely impacted by the pandemic than those without SEND status. What was clear for studies that compared SEND and non-SEND groups for pre-pandemic mental health, was that children with SEND were at a particular risk for poorer mental health even before the exacerbating occurrence of the pandemic, and when COVID-19 struck, the mental well-being of these children lowered further. Vasa et al. (2021) even reported that most of their autistic sample already had a mental health diagnosis prior to the onset of the COVID-19 pandemic. There is some evidence that group differences in mental health/well-being for children with SEND compared to TD children during the COVID-19 are simply due to the typically poorer mental health/well-being of this group prior to the pandemic (e.g. Rzepecka et al., 2011; Van Steensel et al., 2011) and the decline in mental health/well-being for child samples, regardless of SEND status (Kreysa et al., 2022; Sideropoulos et al., 2022). By conducting both a review of the literature and a survey on the effects of COVID-19 on children with autism, Kreysa and colleagues (2022) noted that although autistic children scored lower for emotional functioning than TD children, TD children showed similar decreases in well-being during the pandemic to the autism group. Another study comparing anxiety and worries for children and young adults with SEND and their TD siblings across three time-points before and during the first UK national lockdown found similar increases in anxiety for both SEND youth and their TD siblings (Sideropoulos et al., 2022). Notably, the study further found that awareness of COVID-19, caregiver anxiety, and a previous diagnosis of an anxiety disorder were all significant predictors of youth anxiety at the start of the lockdown for the SEND group (Sideropoulos et al., 2022). This study mirrors findings presented in the current review, suggesting the importance of individual differences, but the overall negative impact of the pandemic on youth mental well-being.

For studies that presented findings suggesting that a child’s understanding of COVID-19 and/or their language ability played a role in the extent of the impact of the pandemic on their mental health or well-being (Bhat, 2021; Vasa et al., 2021), parent-report was relied upon. Bhat (2021) acknowledged that parents of children with more complex communication needs have under-reported the distress that their child actually experienced. Although the issue that parents may not be identifying the extent of their child’s distress is a possible limitation of any parent-report study, there is likely a much greater risk of disparity between a child’s experience and parent’s report when the child is not able to communicate their experience verbally. It is possible that inaccurate reporting of the experience of those with complex communication needs applies across the studies included in the current review, as studies that did use child-report measures did not include children falling into this category. Pellicano et al. (2022) acknowledge that their results may underestimate the distress experienced by all autistic youth as they only included children who could communicate verbally using traditional neuro-normative methods.

### Role of Methodology Choice on Study Findings and Study Quality

Despite most papers included in the current review finding some evidence for a negative effect of the COVID-19 pandemic, studies were less likely to find an effect if they employed a longitudinal design; this may be at least partly due to these longitudinal designs typically using standardized measures, as studies using standardized measures were less likely to report an effect than those using researcher-made tools, particularly when examining anxiety as an outcome. It is debateable whether this difference is due to standardized measures being more reliable, having been previously tested and validated, or if during these unprecedented times, researcher-made measures designed for use in this pandemic were needed to tap into experiences that may have been unique to the pandemic. Equally, any difference may have been due to standardized measures typically measuring trait-level symptoms and researcher-made measures examining state-level symptoms, meaning that standardized measures are not designed to measure smaller or short-term changes. For the studies included in the current review, the analyses reported using researcher-made measures that were piloted and/or validated before use in the study still reported finding negative effects on child mental health. Ultimately, even though studies utilizing standardized measures were less likely to report finding a negative effect on mental well-being, studies were more likely to report a negative effect for at least one sub-group than a positive effect or no effect at all, regardless of whether a standardized or researcher-made measure was chosen.

A greater difference was seen for studies employing either a longitudinal or cross-sectional design, with longitudinal studies less likely to report a significant negative effect on child mental health or well-being. This difference in whether effects were reported depending on methodological design may suggest that retrospective accounts are less accurate and are influenced by the typically poorer mental health of children with SEND, with parents and children both reporting a greater change in mental health or well-being than repeated reports of standardized measures would imply had taken place. Interestingly, several studies indicated that SEND groups experienced poorer mental health at each time-point than typically developing (TD) controls (e.g., Corbett et al., 2021; Raw et al., 2021). It may be then, that when examining mental health or well-being longitudinally it can be difficult to establish an effect due to the already inflated difficulties presented pre-pandemic (Amorim et al., 2020; Corbett et al., 2021; Toseeb & Asbury, 2022; Yarger et al., 2021). Thus, although children with SEND may have reported exaggerated anxiety and poor mood, the difference between pre-COVID-19 and pandemic assessments may be less clear. Equally, it may be that parents struggling with coping during the COVID-19 pandemic over-estimated their child’s mental health struggles. However, it is also possible that the reduced chance of studies reporting an effect when using a longitudinal design was due to the method used to establish whether an effect was seen. The longitudinal studies tended to use null hypothesis significance testing to establish whether a change in mental health/well-being could be asserted, whereas the only cross-sectional studies to use significance testing had to treat the analysis as if it was longitudinal by asking reporters to reflect back on previous symptoms and experiences, before reflecting on current experience. Instead, most cross-sectional studies simply used descriptive statistics to report percentages of families who experienced a negative impact, with no arbitrary cut-off point for when to establish that an impact was apparent. Whether traditional significance testing is too strict to establish a genuine effect in this unique circumstance or whether it is necessary to employ such methods to assess the extent of any potential impact is an issue that we offer for debate. Notably, twice as many studies using descriptive comparisons compared to significance testing reported a negative impact for the child. Equally, 80% of the studies reporting a positive impact also used descriptive methods to report this change. The one paper setting out to explicitly examine change in positive well-being using significant testing found no change in this area of well-being. Where individual differences are likely to play a large role in determining the impact of an event such as a pandemic, perhaps significance testing methods alone cannot truly highlight the challenges that some individuals face during these periods, or equally the challenges that are typically faced. By comparing studies using a variety of techniques we have been able to conclude that the mental well-being of children with SEND status was typically poorer during the pandemic, yet individual differences were evident. Studies using methods other than significance testing were more likely to describe any alleviation of typically experienced problems relating to traditional school settings. It’s also key to note that there is a difference between not finding evidence for an effect and finding evidence for no effect, with studies not reporting an impact of the pandemic potentially not reaching significance levels for an effect, rather than implying a lack of one. Sometimes when samples vary within themselves, significance levels cannot be reached, and as explained above different children with SEND may have had different experiences of the pandemic depending on personal circumstances.

Most studies included in this review opted for parent-only reports of their child’s mental health or well-being. For studies examining the impact of the COVID-19 pandemic on anxiety, this did not lead to a discrepancy between studies based on chosen reporter, yet for mood there was a greater chance of a null effect being reported when parents were asked without children as alternative or co-reporters. Despite parents in one sample reporting that they felt confident that their own reports accurately reflected their child's mental health (Theis et al., 2021), previous research has noted that parent and child reports can vary (Muris et al., 2003), and so consideration should be given for future studies to the reliability of parent-only reports if the child sample is at an appropriate developmental age to provide a self-report. Perhaps, given the potential discrepancy between child and parent report, combined parent and child reports where appropriate could be encouraged.

### Limitations and Future Research

Studies varied in the level of transparency taken, which became especially clear upon commencement of data extraction for this review. Future studies need to be particularly mindful of the importance for full transparency in reporting research. Perhaps research groups should look to following guidelines such as the Hong Kong Principles for assessing researchers (Moher et al., 2020) to ensure that clarity over how research has been conducted and where limitations lie within their studies. For some studies it was less transparent as to how SEND was defined and therefore to account for the role of SEND identifying processes and cultural perceptions of SEND across different countries on the results of the related studies. This limitation should be taken into account when considering the findings of the present study. This review has tried to describe the literature as best as possible given the information that was provided in the studies included (and those rejected).

## Conclusions

Despite the differences in research approach used by researchers, including differences in how mental health/well-being was measured and how change was established, the overwhelming conclusion that can be made upon review of the literature is that the COVID-19 pandemic had an overall negative impact on mental health and well-being for children with SEND, similarly to effects seen for TD groups.

Individual factors did emerge, however, and highlighted that one blanket response to attempt to protect children with SEND may not be helpful, with a number of factors seen as paying a role in the impact that the pandemic played, and the types of stressors that caused problems. Where children with SEND experience typically poorer mental health/well-being than their TD peers, future adverse events may further exacerbate any pre-existing mental health problems. If a similar event such as the COVID-19 pandemic occurs in the future, we need to protect this group from being further disadvantaged, and work to improve this group’s mental well-being post-pandemic. Ensuring that support plans can be put in place for these families would help to reduce the risk of both child and parent mental health deterioration and promote positive mental well-being outcomes group. Such support plans are clearly important, regardless of future global adverse events, and should be flexible enough to accommodate individual differences in support needs; in this instance, when adversity in any form strikes, tailored support can reduce the impact. Equally, individual or smaller-scale causes of distress may also act to add to initial causes of mental distress that many children with SEND already face. Children with SEND status are a group that are at greater risk of mental health problems, and there is a clear need for programs that help to improve the mental well-being of these individuals. Whilst adverse life events can increase the risk of mental health problems for any child, SEND children are a group with typically poorer mental health, and thus the presence of further stressors may lead to a poorer outcome for this group. More research is needed on factors that act as buffers against negative effects on SEND child mental health, and importantly, more is needed to be done to improve every-day mental health for children with SEND. Factors such as the lack of opportunities for socializing with peers face-to-face, loss of routines, and remote learning were considered problematic for some children in the reported studies, yet these same factors actually helped reduce anxiety for other children; therefore, any efforts made to improve the mental health of this group need to be flexible enough to recognize individual differences in causes of distress and mental relief. These lessons generated from the findings of the studies highlighted in this review must now be applied to day-to-day life; a clear effort needs to be made to improve the everyday mental well-being of children with SEND, both for improved day-to-day quality of life and to prevent a deterioration in mental health if adverse life events are ever to occur, with efforts taking a flexible or multifaced approach in order to account for individual differences in support needs. A need for successful mental health interventions specifically targeted at and co-designed with SEND groups is clear. Such interventions are required alongside policy guidelines that take into account findings that some SEND children find traditional education features, such as class-room size and noise-level, challenging. Tailored support packages where feasible would benefit this population immensely. Although it may not be possible to deliver tailored support to every SEND child around the world, what is needed is a recognition that not all suggested ‘solutions’ may act to improve the mental health of each child, and where this is a population that is at increased risk of poorer mental health, a helpful approach may be to routinely screen this population for mental health problems and then provide tailored support (including adjustments to education deliver where appropriate) to those struggling the most. Furthermore, in order to allow children to flourish and experience the best possible developmental outcomes, we need to promote mental well-being opportunities, rather than focus solely on the reduction of clinical mental health diagnoses.

Additionally, researchers need to ensure that high transparency and quality of research is attained for the most reliable and insightful findings to be produced, especially given the difference that different methodological approaches can make to study findings and concluded implications.

## Data Availability

The data that support the findings of this study are all available and can be tracked using the DOIs. However, a full list of the texts and associated materials will be available online here: https://osf.io/u6tdm/.

## Footnotes

1 Here, we considered Türkiye as part of the continent of Asia, as more land is located within Asia than Europe.
